# Evaluating patient-reported outcome measures in Peru: a cross-sectional study of satisfaction and net promoter score using the 2016 EnSuSalud survey

**DOI:** 10.1136/bmjqs-2021-014095

**Published:** 2022-02-04

**Authors:** Hannah H Leslie, Hwa-Young Lee, Brittany Blouin, Margaret E Kruk, Patricia J García

**Affiliations:** 1 Division of Prevention Science, University of California San Francisco, San Francisco, California, USA; 2 Department of Global Health and Population, Harvard University T H Chan School of Public Health, Boston, Massachusetts, USA; 3 Convergence Science Academy, Institute of Convergence Science (ICONS), Yonsei University, Seoul, South Korea; 4 School of Public Health, Universidad Peruana Cayetano Heredia, Lima, Peru

**Keywords:** quality measurement, patient satisfaction, patient-centred care, ambulatory care

## Abstract

**Background:**

Patient-reported measures attempt to quantify the value health services provide to users. Satisfaction is a common summative measure, but often has limited utility in identifying poor quality care. We compared satisfaction and the net promoter score (NPS), which was developed to help businesses quantify consumer sentiment, in a nationally representative survey in Peru. We aimed to compare NPS and satisfaction as individual ratings of care, assess the relationship of patient-reported experience ratings to these outcome measures and consider the utility of these measures as indicators of facility performance based on reliability within facilities and capacity to discriminate between facilities.

**Methods:**

We analysed the 2016 National Survey on User Satisfaction of Health Services, a cross-sectional outpatient exit survey. We assessed ratings by patient characteristics and compared the distributions of satisfaction and NPS categories. We tested the association of patient-reported experience measures with each outcome using multilevel ordinal logistic regression. We used intraclass correlation (ICC) from these models to predict minimum sample for reliable assessment and compared patient-reported experience measures in facilities with average satisfaction but below or above average NPS.

**Results:**

13 434 individuals rated services at 184 facilities. Satisfaction (74% satisfied) and NPS (17% reported at least 9 out of 10) were largely concordant within individuals but weakly correlated (0.37). Ratings varied by individual factors such as age and visit purpose. Most domains of patient-reported experience were associated with both outcomes. Adjusted ICC was higher for NPS (0.26 vs 0.11), requiring a minimum of 7 (vs 20) respondents for adequate reliability. Within the 70% of facilities classified as average based on satisfaction, NPS-based classification revealed systematic differences in patient-reported experience measures.

**Conclusion:**

While satisfaction and NPS were broadly similar at an individual level, this evidence suggests NPS may be useful for benchmarking facility performance as part of national efforts in Peru and throughout Latin America to identify deficits in health service quality.

## Introduction

Meeting population needs and expectations for health services is a core function of health systems: high-quality health systems should produce satisfied patients.^1^ Patient satisfaction reflects whether the care received has delivered as individuals expect, and as such can be used to hold providers, facilities and regions accountable for health system performance.^2^ In Latin American countries, which have championed the right to health and the pursuit of universal health coverage,^3^ assessment of patient satisfaction and endorsement of the health system is a priority across and within countries.^4–6^ Given the cost of such assessments and the increasing demands on health system financing, identifying a minimum set of measures of health system performance that are responsive to population preferences and can direct and inform improvement efforts is a high priority.^7^

Peru is a middle-income country of over 32 million people that, prior to the COVID-19 pandemic, experienced substantial gains in life expectancy despite a health workforce shortage and increasing burden of non-communicable diseases.^8^ The government of Peru conducted surveys of patient experience and user satisfaction with outpatient care from 2014 to 2016. Analyses of these data have identified generally high satisfaction, with lower satisfaction in individuals waiting longer for care or experiencing shorter consultations.^9^ These analyses and others recognised the limitations of satisfaction as a measure of health service quality, particularly the high levels of satisfaction documented in the presence of poor quality care.^1 10^ Responses to satisfaction questions require individuals to transform experiences into overall evaluations, a process that depends on prior expectations for care and the degree to which individuals hold healthcare providers responsible for any deficits in care.^2 11^ Expectations of care will be shaped by individual and contextual factors, including educational attainment, social status, previous healthcare experience and low agency or disempowerment relative to healthcare providers.^2 12^ Low expectations of care quality reduce the sensitivity of satisfaction measures to identify inadequate or low-quality health services; evidence from an internet survey in 12 low-income and middle-income countries found that approximately half of respondents had low expectations for the technical and interpersonal quality of care received.^12^

An alternative to patient satisfaction measures that still provides a summative assessment using a single item is the net promoter score (NPS), initially proposed as a metric of brand performance in 2003^13^ and used extensively by businesses conducting customer surveys since then. The NPS asks, ‘How likely are you to recommend this service to your friends and family?’ and can be summarised within services or facilities to compare those promoting and disparaging the service. The NPS and satisfaction differ in the focus of the item—a potential future recommendation versus expectations having been met, the span of response options (typically 10 for NPS and 3–5 for satisfaction) and the intended use, as NPS originated specifically as an aggregate measure with a calculation that emphasises responses near the extremes. Only responses of 9 or 10 are considered positive. The NPS could offer an improvement over satisfaction for rating health services if it demonstrates at least comparable sensitivity to content of the visit^14^ and enables differentiation between better and worse health services or facilities to inform corrective action. The NPS has been used to evaluate health services in several studies in high-income settings, including a large assessment in Dutch health facilities identifying correlation between NPS and measures of patient experience and satisfaction, but no evidence for specific added value of the measure.^15^ A modified 5-point version of NPS was deployed in the National Health Service in England as the Friends and Family Test, intended originally to identify best performing providers and later to inform local quality improvement within general practice offices.^16^ Studies of this measure with specific patient populations found reasonable reliability^17^ and correlation with functional improvement after surgery.^18^ However, in general inpatient and outpatient settings, utility of the Friends and Family Test for ongoing quality improvement was limited by low response rates, systematic differences in responses based on mode of administration and demographic characteristics, and lack of specificity to inform practice-level improvements.^16 19^

Little is known on the utility of the NPS for health services outside of high-income settings. In many middle-income countries, including Peru, cross-sectional health facility assessments have been used to benchmark health service performance and inform policy, with satisfaction the primary outcome measure.^1 7 20^ While both satisfaction and NPS were asked in some form in health facility assessments in Peru from 2014 to 2016, survey reports focused only on satisfaction.^21^ An alternative to satisfaction could add value in this context if it accords with patient perspectives on the experience of care, helps distinguish good services from services meeting low expectations to target top-down improvements and can be administered in such surveys or in lighter touch assessments.

In this analysis, we use detailed assessments of patient experience and satisfaction with health services in Peru to test whether the NPS is a valid and useful measure of health service quality at the individual level and facility level in this setting. We aim specifically to compare NPS and satisfaction as individual ratings of care, assess the relationship of patient-reported experience ratings to satisfaction and to NPS and consider the utility of satisfaction and NPS as indicators of facility performance based on reliability within facilities and capacity to discriminate between facilities.

## Methods

### Setting

Health services in Peru are provided in the public sector by the Ministry of Health (MoH) to the population that lacks insurance and/or is living in poverty, by the Ministry of Labor through the EsSalud programme to formally employed individuals and their close family members and by the armed forces and national police forces. The private sector is financed by private insurance or out-of-pocket payment.^22^ As of 2015 when the patient satisfaction surveys were being conducted regularly, 37% of the population was covered by MoH, 21% by EsSalud, 3% within armed forces and 5.5% with private insurance, leaving just over 37% without insurance^23^; public insurance coverage has since been expanded.^8^

### Data sources

For this secondary analysis, data were taken from the National Survey on User Satisfaction of Health Services (EnSuSalud).^24^ EnSuSalud was developed and piloted in 2014 and conducted in 2014, 2015 and 2016 by the National Institute of Statistics and Informatics (INEI in Spanish) in collaboration with the National Superintendency of Health in Peru.^20 21 25^ EnSuSalud is composed of six modules; we used the module administered to patients following outpatient consultations and conducted this analysis on the 2016 survey as the most recent year available (2014 and 2015 surveys included slightly different patient-reported experience and outcome items).

The EnSuSalud survey was a cross-sectional assessment of health system users interviewed at health facilities; full details on development and administration are available from INEI.^21^ Briefly, a probabilistic, stratified two-stage and independent sampling was adopted. A master facility list of all formal health facilities in Peru was stratified by type (MoH, EsSalud insurance, armed forces and police, private); health facilities were selected within strata with probability proportional to daily visit load. In the second stage, outpatient consultation users aged ≥15 years were selected using systematic random sampling and approached to participate by trained pollsters. No incentive was offered to participants to respond to the survey. Sample size was calculated to provide sampling error of up to ±5% for patient satisfaction within each subnational region.^21^ Data for this analysis were collected in the 2016 survey (May to July 2016). Sampling weights were calculated for each facility; we rescaled weights to total to the analytical sample. All items in EnSuSalud were administered in Spanish; one author (HHL) translated items to English with review by two authors fluent in Spanish (PJG and BB).

We drew supporting data from INEI on poverty in 2009, the most recent year census-based data were available.^26^

### Measurements: patient satisfaction and NPS

Patient satisfaction was measured using the item, ‘Regarding the service you received today at this facility, how would you rate your level of satisfaction?’ with 5-point response options ranging from 5 ‘very satisfied’ to 1 ‘very dissatisfied’. We analysed the original response scale and a three-category version of satisfied (satisfied or very satisfied), neutral and not satisfied (very dissatisfied or dissatisfied). The number of categories was selected to match the NPS classification and the groupings determined based on the INEI definition of satisfaction as a primary outcome of EnSuSalud.^21^ As a sensitivity analysis, we reclassified ‘satisfied’ as neutral and ‘neither satisfied nor dissatisfied’ as not satisfied to mimic the numeric classifications of the NPS.

The NPS item read: ‘If you had to recommend the services of this health facility, what score would you give it on a scale of 1 to 10, where 1 is never and 10 is always?’ We used this 10-point NPS, designated NPS_10_, and a three-category version based on established values: 1–6 for ‘detractors’, 7 and 8 for ‘passives’ and 9 and 10 for ‘promoters’.^15 18^

### Independent variables

We identified covariates at contextual, facility, individual and visit levels based on previous analyses of patient satisfaction.^10 14 27^ Contextual variables included the region (Coast, Jungle, Andean and Metropolitan Lima) and the percentage of the population in poverty of the district where the facility is located from the 2009 census.^26^ Facility factors included type (MoH, EsSalud insurance, armed forces and police, private) and level (primary, secondary (small hospital) and tertiary (referral hospital)). For individual-level factors, we included sociodemographic characteristics, health status and visit attributes. Sociodemographic characteristics included patient age (classified into <30, 30–44, 45–59 and ≥60), gender, wealth quintile of the respondent based on an asset index comparable to the index used in Demographic and Health Surveys and education level (grouped from 11 response options into four: less than primary, completed primary (6 years of elementary education), some secondary or completed secondary, some or completed higher education—university or non-university^28^). Health status was measured using self-rated health, measured on a scale of 0 (worst imaginable) to 100 (best imaginable). Original response options were confined to multiples of 5 so we recoded this to 0–20 for ease of coefficient interpretation. Visit attributes included type (referral from external facility, referral within facility, recurring visit and first visit) and purpose (existing disease, new disease, antenatal care, medical check-up and for addressing discomfort, pain, fever, etc, without a diagnosis). For indicators of service quality at the visit level, we applied the framework from the *Lancet Global Health* Commission on High-Quality Health Systems^1^ to define process quality within domains of user experience and competent care. One author identified candidate items among the patient-reported experience ratings in EnSuSalud (H-YL) and two authors reviewed and confirmed salience per domain (HHL and PJG). Items were mapped to seven domains: dignity, communication, privacy, wait time, ease of use, provider competence and timely action (see online supplemental table S1). We generated standardised scores (mean 0, SD 1) based on all indicators within each subdomain.

10.1136/bmjqs-2021-014095.supp1 Supplementary data

### Analyses

To increase the comparability of all results, we used the three-category versions of satisfaction and NPS as the primary outcomes for all analyses. We limited the analysis to respondents with complete data and with visits for preventive or curative care (excluding coming for health certificates only or for unclassified reasons). First, we reported descriptive statistics on characteristics of the individuals and facilities of the analytical sample. We calculated per cent satisfied in the three-category version of satisfaction, average NPS_10_, and per cent promoters by each covariate to identify differences in ratings between respondents. Then, we assessed the relationship of satisfaction and NPS at the individual level by showing the distribution of responses on each outcome overall and by category of the other measure (for instance, the proportion of promoters, passives and detractors for each satisfaction response option). We calculated Spearman rank correlation for the original versions of satisfaction and NPS_10_. These descriptive analyses incorporated survey sampling weights. Subsequent analyses are unweighted based on inclusion of the study design factors such as facility management type, level and region in the analysis.

We conducted two analyses to understand the association of patient-reported experiences with satisfaction and NPS. First, we modelled the categorical version of each outcome on all domains of patient-reported experience using multilevel ordinal logistic regression. Models were adjusted for contextual, facility and individual factors and tested for collinearity using linear regression and considering variance inflation factor <4.0 acceptable. Second, to provide an overall assessment of whether the models for satisfaction and NPS are improved by adding patient-reported experience, we used ordinal logistic regression models clustered by facility for the three-category version of each outcome and reported the pseudo-R^2^ from models with contextual, facility and individual factors and then with the addition of patient-reported experience. We repeated these analyses as a linear regression with NPS_10_ as the outcome and used R^2^ to quantify variance explained by the addition of patient-reported experience variables.

We next assessed satisfaction and NPS within facilities. First, we calculated the intraclass correlation (ICC) as a measure of the extent to which individual ratings can be explained by facility; it is calculated as the proportion of observed variance in ratings that is due to systematic between-facility difference compared with the total variance in ratings. ICC ranges from 0 to 1; higher values indicate greater clustering of ratings within relative to between facilities. We considered 0.05 as the minimum indication of a facility effect.^29^ We calculated ICC from multilevel ordinal logistic regression models for each categorical outcome and multilevel linear regression for NPS_10_, first as null models and then controlling for individual-level factors (demographics, self-rated health and visit type and purpose) to address case-mix differences between facilities. We used the Spearman-Brown formula, $\frac{\left(n*ICC\right)}{\left(1+\left(n-1\right)*ICC\right)}$ , where n is sample size per facility, to calculate reliability and estimate the minimum number of respondents required per facility to obtain adequate (>0.70) or strong (>0.90) reliability for each outcome.^30^

Second, to show discrimination between facilities, we generated facility-specific means (empirical Bayes estimates) and SEs from the multilevel ordinal logistic regression models of each outcome adjusted for case mix. We calculated the Spearman rank correlation for facility scores on satisfaction and NPS. We then plotted these estimates with 95% CIs and identified facilities as below average, average or above average on each outcome based on whether the CI included zero. We compared these classifications between satisfaction and NPS. To assess the meaning of differences in classification, we averaged patient experience ratings within facility for the subdomains defined previously. We focused on facilities classified as average using satisfaction and compared mean patient-reported experience ratings across facilities classified as below average, average or above average using NPS. We reported mean experience ratings for these classifications and tested group-level difference using one-way analysis of variance. All analyses were conducted in Stata V.17.0 (StataCorp, College Station, Texas).

## Results

### Survey responses

Of 14 110 individuals approached to participate in EnSuSalud, 13 814 consented and completed the survey (97.9%) with the remainder refusing (2%) or not completing the full survey (0.1%).^21^ Individuals who visited the health facility only for receiving health certificates (n=75) or for other unclassified purposes (n=293, including checking examination results or postoperative review) and those with missing data (n=12) were excluded from this analysis. These individuals were more likely to be male, young, from the coastal or mountain region and either first visit or outside referrals (online supplemental table S2).

### Individual-level analysis

The final analytical sample is composed of 13 434 individuals from 184 facilities, with a median of 60 respondents per facility (IQR 35–101, minimum 4). Most users were from MoH (raw n=6309) and EsSalud (5934) facilities, along with 513 users of military/police services and 678 seeking private services. Most respondents were female (60.5%); 19% were aged 60 and above, and over 40% had at least some higher (postsecondary) education (table 1). Overall, patient ratings were moderately high: 74% of respondents were at least satisfied with services received, while the average NPS_10_ was 7.06, with 17% of respondents classified as promoters. Both measures showed differences based on demographics and visit type that support case-mix adjustment when comparing across facilities: ratings on each measure were higher among users in older age categories and in higher wealth quintiles and lowest for those with a new disease. In summarising nationally, the highest ratings were observed in Lima (77% at least satisfied, 24.7% promoters). Within facilities, ratings were the highest in private facilities (average of 89% of respondents satisfied, 33% promoters). Differences by facility level were less substantial, although both ratings were lowest in primary facilities.

**Table 1:** Descriptive statistics of the analytical sample

	Individualsn (%)	Satisfaction with services	Facility recommendation	
		At least satisfied n (%)	NPS_10_ Mean (SD)	Promoters n (%)
Total	(n=13 432)	9925 (74)	7.06 (1.64)	2320 (17)
Gender				
Male	5311 (39.5)	4029 (75.9)	7.09 (1.61)	880 (16.6)
Female	8122 (60.5)	5896 (72.6)	7.05 (1.65)	1440 (17.7)
Age categories (years)				
<30	4113 (30.6)	2866 (69.7)	6.94 (1.63)	640 (15.6)
≥30 and <45	3606 (26.8)	2595 (72.0)	7.00 (1.68)	629 (17.4)
≥45 and <60	3121 (23.2)	2360 (75.6)	7.16 (1.68)	639 (20.5)
≥60	2593 (19.3)	2106 (81.2)	7.23 (1.51)	414 (15.9)
Region				
Costa (Coast)	3278 (24.4)	2389 (72.9)	6.72 (1.47)	311 (9.5)
Selva (Jungle)	3104 (23.1)	2202 (70.9)	6.75 (1.60)	334 (10.7)
Sierra (Mountain)	1283 (9.5)	894 (69.7)	7.02 (1.94)	252 (19.6)
Metropolitan Lima	5769 (42.9)	4441 (77.0)	7.44 (1.59)	1425 (24.7)
Wealth quintile				
1st (poorest)	2119 (15.8)	1446 (68.2)	6.87 (1.60)	285 (13.4)
2nd	2238 (16.7)	1483 (66.3)	6.83 (1.63)	323 (14.4)
3rd	2395 (17.8)	1700 (71.0)	6.88 (1.62)	328 (13.7)
4th	2612 (19.4)	1917 (73.4)	6.95 (1.56)	347 (13.3)
5th (wealthiest)	4071 (30.3)	3381 (83.0)	7.48 (1.63)	1039 (25.5)
Education level				
<Primary	1044 (7.8)	743 (71.2)	7.02 (1.57)	162 (15.5)
Completed primary	1007 (7.5)	782 (77.7)	7.21 (1.69)	211 (20.9)
Some/completed secondary	5563 (41.4)	4007 (72.0)	7.00 (1.62)	879 (15.8)
Some/completed higher education	5819 (43.3)	4394 (75.5)	7.11 (1.65)	1069 (18.4)
Purpose of visit				
Existing disease	5905 (44.0)	4424 (74.9)	7.14 (1.62)	1097 (18.6)
New disease	2963 (22.1)	2029 (68.5)	6.84 (1.59)	381 (12.9)
Pregnancy check	606 (4.5)	463 (76.3)	6.99 (1.55)	105 (17.2)
Medical check	2610 (19.4)	2022 (77.5)	7.26 (1.64)	519 (19.9)
Discomfort, pain, fever	1351 (10.1)	989 (73.2)	6.87 (1.75)	220 (16.3)
Type of visit				
Outside referral	1443 (10.7)	1060 (73.4)	6.94 (1.60)	157 (10.8)
Internal referral	528 (3.9)	392 (74.2)	6.99 (1.74)	72 (13.6)
Recurring visit	6952 (51.8)	5206 (74.9)	7.14 (1.63)	1366 (19.6)
First visit	4511 (33.6)	3270 (72.5)	6.99 (1.63)	726 (16.1)

	Facilities (n=184)	Satisfaction with services	Facility recommendation	
		Proportion satisfied per facility Mean (SD)	NPS_10_ Mean (SD)	Proportion of promoters per facility Mean (SD)
Facility type				
Ministry of Health	79 (43%)	0.66 (0.16)	6.85 (0.72)	0.13 (0.13)
EsSalud insurance	50 (27%)	0.82 (0.16)	7.27 (0.72)	0.14 (0.14)
Armed forces and police	16 (8%)	0.80 (0.10)	7.25 (0.53)	0.20 (0.09)
Private	40 (22%)	0.89 (0.11)	7.90 (0.54)	0.33 (0.16)
Facility level				
Primary	83 (45%)	0.72 (0.18)	7.03 (0.77)	0.17 (0.15)
Secondary	79 (43%)	0.83 (0.15)	7.41 (0.78)	0.18 (0.16)
Tertiary	23 (12%)	0.73 (0.14)	7.30 (0.67)	0.23 (0.14)

Weighted summaries; frequencies may not sum exactly to total due to rounding.

NPS, net promoter score.

**Figure 1:**
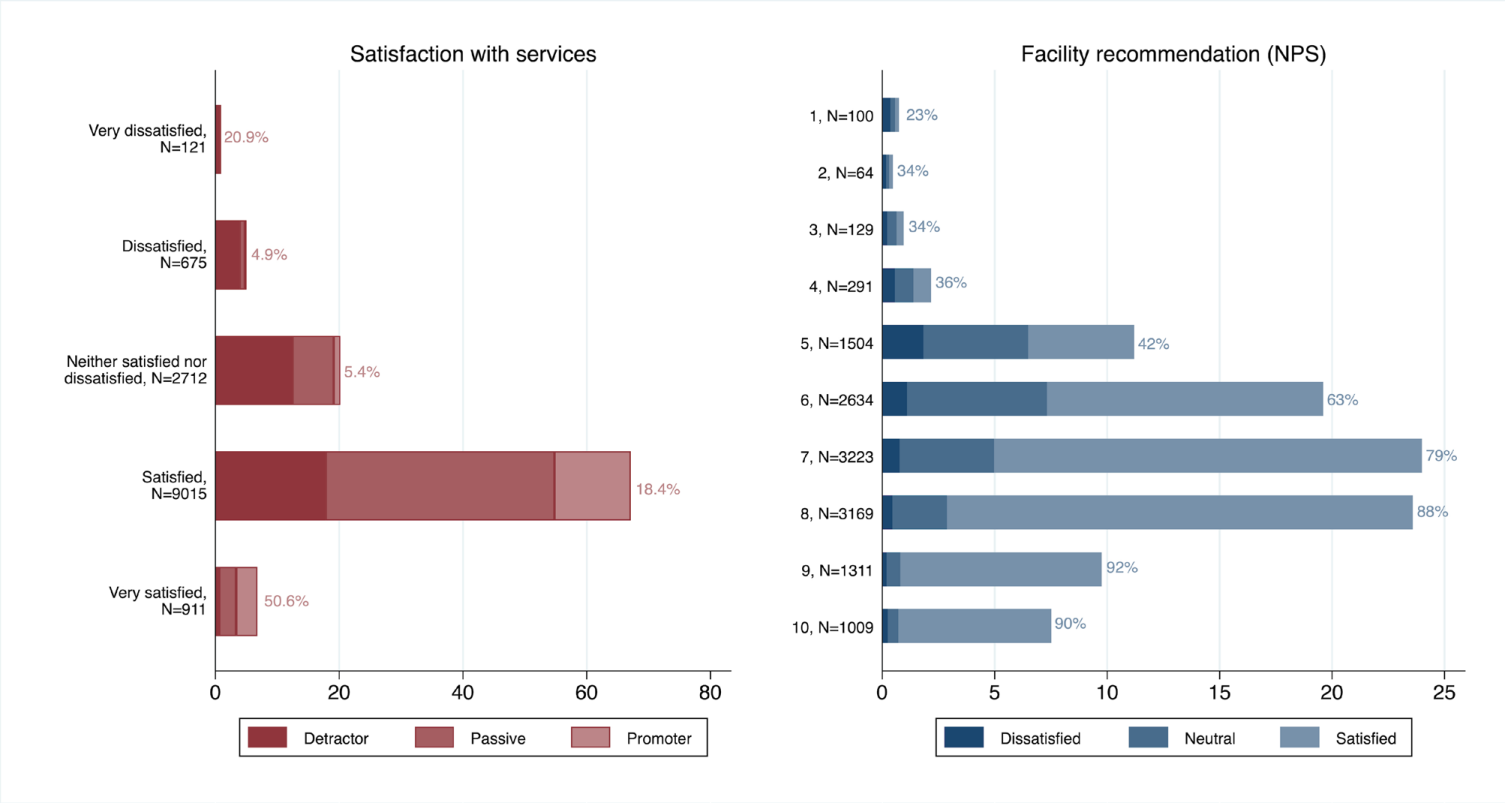
Distribution of patient outcome-reported measures for satisfaction and facility recommendation (NPS_10_). Histograms show per cent of respondents in each response category for satisfaction and facility recommendation. Bar shading shows the fraction of respondents per bar in categories of the other measure, for instance, fraction of those very satisfied classified by the NPS as promoters (50.6%) and fraction of those who chose 10 on NPS_10_ classified as satisfied (90%). NPS, net promoter score.

Figure 1 displays the distributions of the two measures overlaid with the categorical version of the alternative measure. Satisfaction responses were less broadly distributed than NPS_10_ responses, with two of every three respondents selecting ‘satisfied’. Reporting being very satisfied or any level of not satisfied was less common than selecting a corresponding level on NPS_10_: 6.8% of respondents were very satisfied compared with 17.3% promoters (NPS_10_ ≥9), 26.1% were less than satisfied compared with 35.2% detractors (NPS_10_ ≤6). Aside from the small very dissatisfied and NPS_10_=2 categories, the general direction of the two measures was consistent, with increasing per cent of promoters at each increasing level of satisfaction and increasing per cent of those at least satisfied as NPS_10_ increased. Findings were similar for the alternative classification of satisfaction (online supplemental figure S1). Spearman correlation between NPS_10_ and 5-point satisfaction score was weak at 37%.

Table 2 shows the associations of patient-reported experience domains with categorical versions of satisfaction and NPS in the fully adjusted multilevel ordinal logistic regression models (full models and models for alternative outcome classifications shown in online supplemental table S3). Neither model showed evidence of collinearity. After adjustment for contextual, facility and individual factors, most elements of patient-reported experience were positively associated with both ratings, with the strongest associations for dignity and communication with satisfaction and for communication and especially ease of use with NPS. For each 1 SD increase in communication rating, the odds of selecting a higher category of satisfaction were 2.73 times greater and the odds of selecting a higher category of NPS rating were 2.02 times greater. Ratings of dignity and wait time were associated only with satisfaction; privacy ratings were associated only with NPS. Ratings of provider competence were not associated with either outcome measure. The addition of patient-reported experience domains increased the pseudo-R^2^ from 0.03 to 0.20 for satisfaction and from 0.04 to 0.21 for NPS (online supplemental table S4). The pattern of association was similar for alternative classifications of each outcome; the increase in variance explained with the addition of patient ratings of the visit was substantial in linear regression of NPS_10_ (R^2^=0.08 to 0.37).

**Table 2:** Association of patient-reported experience domains with satisfaction and facility recommendation (n=13 434)

	Satisfaction with service (satisfied, neutral, dissatisfied)		Facility recommendation NPS (promoters, passive, detractors)	
	AOR	95% CI	AOR	95% CI
Patient-reported experience (standardised score)				
Dignity	**1.77**	**1.60 to 1.95**	1.05	0.96 to 1.16
Privacy	0.97	0.92 to 1.03	**1.10**	**1.04 to 1.16**
Communication	**2.73**	**2.47 to 3.02**	**2.02**	**1.83 to 2.23**
Short wait time	**1.21**	**1.15 to 1.28**	**1.06**	**1.00 to 1.11**
Ease of use	**1.27**	**1.15 to 1.41**	**3.84**	**3.46 to 4.25**
Provider competence	1.04	0.95 to 1.14	0.94	0.86 to 1.03
Timely action	**1.11**	**1.05 to 1.17**	**1.18**	**1.12 to 1.24**

Bold denotes p<0.05. Associations adjusted for the contextual, facility and individual characteristics shown in table 1.

AOR, adjusted OR; NPS, net promoter score.

### Facility-level analysis

The extent to which patient ratings can be explained by facility is shown in table 3. ICCs are indicative of a modest group effect for both ratings in original and categorical form, with higher ICCs for NPS than for satisfaction. Using the ICC adjusted for individual-level factors to address case mix, at least 20 and 75 respondents per facility would be required for adequate and strong reliability of categorical satisfaction, respectively, compared with 7 and 26 for categorical NPS. Nearly all facilities in the study sample had sufficient sample to provide adequate or strong reliability on the categorical NPS measure.

**Table 3:** Reliability of patient-reported outcome measures per facility

	Satisfaction		Facility recommendation (NPS)	
	Original 5-point	Categorical (dissatisfied, neutral, satisfied)	Original 10-point	Categorical (detractor, neutral, promoter)
ICC, unadjusted	10.7%	12.4%	16.9%	25.8%
ICC, patient mix adjusted	8.8%	10.8%	16.9%	25.9%
**Facility reliability**	**n (%)**	**n (%)**	**n (%)**	**n (%)**
Inadequate (<0.70)	39 (21.2)	22 (12.0)	4 (2.2)	1 (0.5)
Adequate (0.70–0.89)	90 (48.9)	87 (47.3)	60 (32.6)	38 (20.7)
Strong (>0.90)	55 (29.9)	75 (40.8)	120 (65.2)	145 (78.8)

ICC, intraclass correlation; NPS, net promoter score.

**Figure 2:**
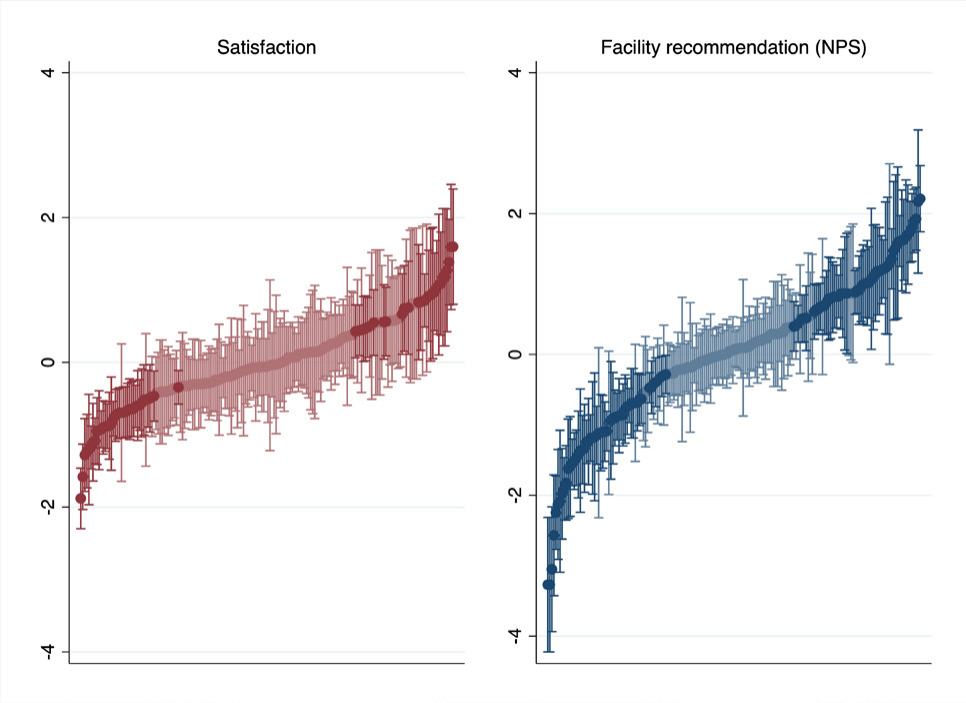
Estimated facility ratings using satisfaction and facility recommendation (NPS). Estimated facility ratings (facility-level residuals from grand mean) and 95% CIs from multilevel models of categorical outcome measures adjusting for individual-level factors. Darker lines indicate facilities statistically below or above average (95% CI excludes 0.0). NPS, net promoter score.

Figure 2 depicts within-facility and between-facility variance on categorical satisfaction and NPS measures based on empirical Bayes predictions from the multilevel ordinal logistic model adjusting for individual-level factors. Correlation of facility scores for satisfaction and NPS was moderate at 58%. The more narrow distribution of satisfaction persisted at facility level: 127 facilities were indistinguishable from average based on categorical satisfaction (69%) compared with 79 (43%) for NPS.

Of the 129 facilities classified as average based on satisfaction, only 68 were also classified as average based on NPS. Patient-reported experience measures of these facilities are shown in table 4 grouped by NPS-based classification.

**Table 4:** Patient ratings of facilities classified as average using satisfaction (n=129)

	Below average NPS (n=24)	Average NPS (n=68)	Above average NPS (n=37)	P value
	Mean (SD)	Mean (SD)	Mean (SD)	
Dignity	−0.04 (0.23)	0.05 (0.34)	0.26 (0.34)	0.001
Privacy	−0.29 (0.47)	0.09 (0.34)	0.43 (0.35)	<0.001
Communication	−0.12 (0.22)	0.07 (0.27)	0.32 (0.29)	<0.001
Wait time	−0.33 (0.41)	0.08 (0.39)	0.37 (0.39)	<0.001
Ease of use	−0.34 (0.34)	0.10 (0.31)	0.44 (0.31)	<0.001
Competence	−0.04 (0.24)	0.05 (0.33)	0.21 (0.32)	0.006
Timely action	−0.14 (0.39)	0.17 (0.40)	0.42 (0.44)	<0.001

NPS, net promoter score.

Patient experience ratings differed substantially and in a direction consistent with the classification based on NPS, increasing as the NPS classification increased from below average, to average, to above average. Findings for the facility-level analyses were similar using the alternative classification of satisfaction (online supplemental figure S2 and table S5).

## Discussion

In this analysis of over 13 000 outpatient surveys in Peru, we found that individual reports of satisfaction and facility recommendation (NPS) were generally consistent; both were associated with most patient-reported experience measures. After classifying each measure to have three response categories, NPS demonstrated higher reliability within facilities and broader range across facilities than satisfaction: this measure showed promise in distinguishing facilities with better or worse patient experience ratings among those classified as average based on satisfaction alone. While single-item patient ratings such as satisfaction and NPS are not a substitute for nuanced patient experience measurement, these findings suggest that in the context of health services in Peru, assessing the NPS in relatively small samples of at least seven patients per facility could provide reliable information to identify better from worse rated facilities to target facility-based interventions.

Both satisfaction scores and NPS varied systematically by individual characteristics and type of visit, a finding in common with use of patient-reported outcome measures in other settings.^19^ Case-mix adjustment to incorporate individual and visit information is important in comparing across groups such as services, supporting the inclusion of at least basic individual information in future assessments of these measures. Both measures were associated with multiple domains of patient-reported experience measures, with substantial unexplained variance in fully adjusted models (roughly 80% for categorical outcomes and 60% for NPS_10_) indicating that observed characteristics and patient experience measures are important but incomplete determinants of summative ratings. The association with communication was strong for both measures, with a stronger link for measures of dignity with satisfaction and ease of use with NPS. This could suggest individuals reflect on how respectfully they were treated in assessing whether services satisfied their expectations but more on convenience in recommending services to others. The only domain not associated with either outcome measure was rating of provider competence, which may suggest a limited contribution of provider technical quality as distinct from elements such as clear communication in summative ratings. Previous studies in many settings have supported the link between patient experience measures and satisfaction,^14 27 31^ including analysis of prior years of EnSuSalud^9^ as well as patient satisfaction surveys in sub-Saharan African countries.^32^ Prior assessment of patient-reported experience ratings and NPS is sparse; one large study among Dutch patients found correlation below 0.40 for a range of patient experience variables and NPS,^15^ with the relationship strongest for communication with healthcare providers. While patient-reported experience measures do not fully explain either satisfaction or NPS, our findings attest that both outcome measures are linked to more granular experiences of health services.

The findings and implication of this study are shaped by the fact that the EnSuSalud survey’s inclusion of both satisfaction and NPS occurred in a context distinct from much of their use in high-income settings: measures were collected in person from a cross section of outpatients across the health system, with a high response rate and towards a primary purpose of benchmarking overall health system performance. While responses to both measures were broadly consistent, the correlation of 0.37 was lower than the correlation between satisfaction and NPS in two studies among English patients, studies in which the numeric scales for satisfaction and NPS matched exactly.^17 18^ Whether due to the difference in concept (satisfaction vs recommendation) and/or the broader range of response options in the NPS, this item did introduce variation within satisfaction categories: while two out of three respondents selected ‘Satisfied’, one-third of these individuals fell into the detractor category of NPS. This range carried through to facility ratings, with 69% of facilities indistinguishable from average using satisfaction compared with 43% for NPS. Patient-reported experience measures differed substantially within facilities grouped as average on satisfaction and in the same direction as NPS classifications. These findings were robust to how satisfaction responses were classified. Combined with the finding that NPS could be reliably assessed with only seven respondents per facility, this evidence suggests that NPS could prove useful, for instance, to the MoH when working to prioritise facilities for intervention given limited resources.

Study findings should be interpreted in light of several limitations. This analysis does not provide evidence of either rating as an outcome or proxy for better clinical care or other objective measures of health service quality. Further analysis testing the relationship between patient-reported measures and clinical outcomes as well as subsequent care utilisation in Peru would help support the broader adoption of patient ratings and the choice of metric in this context. We assessed the capacity of each measure to discriminate between facilities based on the significance of differences in facility ratings given the available sample size and evidence that such differences corresponded to patient-reported experience measures^33^; we are unable to address whether observed differences in ratings between facilities correspond to meaningful differences in care outcomes. This analysis is limited to health service users in Peru in 2016; findings may not generalise to other settings and as the use of health services and methods of assessment continues to evolve. We employed the thresholds for NPS promoter and detractor categories developed in the USA; interpretation of each response value may not be consistent across settings. While the EnSuSalud survey includes a rich set of patient ratings optimised for the Peruvian context,^6^ we could not assess all process of care domains or directly measure user expectations of care.^12^

This analysis is one of the first with the sample size and scope to consider both satisfaction and NPS at the facility level. The findings provide evidence of the capacity of NPS to identify facilities in particular need for improvement, if not to provide detailed guidance on directions for improvement. Although the EnSuSalud survey has been discontinued, methods of data collection such as shorter in-person assessments or even brief mobile surveys^34–36^ that include individual characteristics and measures such as NPS could offer promise as inexpensive population assessments in Peru and elsewhere if measure performance is found to be comparable. Further efforts to combine the use of NPS with objective measures of clinical and system competence could better enable policy makers to identify where health services are failing to deliver to the population in need.

## Data Availability

Data are available in a public, open access repository. Data for this analysis are publicly available from INEI (http://iinei.inei.gob.pe/microdatos/ and http://portal.susalud.gob.pe/blog/category/base-de-datos/). Programming code is available at https://osf.io/mrkuc/.
