# Supplementary material for: Evaluating patient-reported outcome measures in Peru: a cross-sectional study of satisfaction and net promoter score using the 2016 EnSuSalud survey
Source: BMJ Qual Saf. 2022 Feb 4;31(8):599–608. doi: 10.1136/bmjqs-2021-014095 (PMC9304114; doi:10.1136/bmjqs-2021-014095)
Supplement: Supplementary data [file bmjqs-2021-014095supp001.pdf]

Table S1. Items in EnSuSalud used to create patient experience ratings within domains of user experience and competent care and systems.

<table>
<tr><td colspan="2">High-quality health system domains</td><td>EnSuSalud item</td><td></td></tr>
<tr><td>Component</td><td>Sub-component</td><td>Component</td><td>Calculation and Cronbach’s α</td></tr>
<tr><td colspan="4">Positive user experience</td></tr>
<tr><td rowspan="2">Respect</td><td>Dignity (9 items)</td><td>How would you rate on a scale of 1 to 5:
- Administrative staff:
o kindness and courtesy
o respect
o interest in you and willingness to attend
- Non-medical personnel (nurses, laboratory technician, pharmacy technicians, etc)
o kindness and courtesy
o respect
o interest in you and willingness to attend
- Doctors
o kindness and courtesy
o respect
o interest in you and willingness to attend</td><td>Standardized scales were generated for each 3-item set and used to create one overall standardized scale.
• α admin: 0.91
• α non-medical: 0.91
• α doctors: 0.94
• α overall: 0.64</td></tr>
<tr><td>Privacy (1 item)</td><td>· How would you rate the privacy of care on a scale of 1 to 10?</td><td>Item responses were standardized</td></tr>
</table>

	Clear communication (4 items)	· How would you rate on a scale of 1 to 5:<br>- clarity of information provided by non-medical assistance personnel<br>- clarity of information provided by doctors<br>How would you rate on a scale of 1 to 10:<br>- the information provided about your health status<br>- how clearly the treatment and guidelines were explained	Responses to these 4 items were standardized into a scale.<br>α: 0.73
User focus	Short wait times (1 item)	· How would you rate the waiting time for care on a scale of 1 to 10?	Item responses were standardized
	Ease of use (11 items)	· How would you rate on a scale of 1 to 5:<br>o clarity of information provided by administrative staff<br>· How would you rate on a scale of 1 to 10:<br>- location of the facility<br>- compliance with medical care hours<br>- convenience and comfort of the environment of the facility<br>- accessibility of the facility<br>- seats or waiting area of the facility<br>- cleaning – hygiene<br>- signage and orientation posters of the facility<br>- health infrastructure of the facility<br>- administrative procedure of the facility<br>- attention of the administrative staff	Responses to 11 items were standardized into a scale.<br>α: 0.89

Competent care and systems			
Evidence-based, effective care	Provider competence (2 items)	· How would you rate on a scale of 1 to 5:<br>- confidence and security inspired by non-medical personnel<br>- confidence and security inspired by your doctor	Responses to 2 items were standardized into a scale.<br>α: 0.48
	Timely action (1 item)	· How would you rate on a scale of 1 to 10 the time that passed from when you requested the appointment to the date of consultation?	Item responses were standardized

Table S2: Characteristics of excluded observations

	Excluded (N = 382)	Included (N = 13432)
**Gender**		
Male	148 (52.2%)	5350 (39.5%)
Female	136 (47.8%)	8182 (60.5%)
**Age categories**		
<30 years	95 (33.4%)	4144 (30.6%)
>=30 & <45 years	76 (26.7%)	3633 (26.8%)
>=45 & <60 years	64 (22.3%)	3144 (23.2%)
>=60 years	50 (17.6%)	2613 (19.3%)
**Region**		
Costa (Coast)	84 (29.4%)	3303 (24.4%)
Selva (Jungle)	73 (25.7%)	3127 (23.1%)
Sierra (Mountain)	42 (14.5%)	1292 (9.5%)
Metropolitan Lima	87 (30.4%)	5811 (42.9%)
**Wealth quintile**		
1st (poorest)	51 (17.8%)	2134 (15.8%)
2nd	51 (17.8%)	2254 (16.7%)
3rd	51 (17.7%)	2413 (17.8%)
4th	56 (19.6%)	2631 (19.4%)
5th (wealthiest)	78 (27.2%)	4101 (30.3%)
**Education level**		
< Primary	18 (6.3%)	1052 (7.8%)
Completed primary	21 (7.4%)	1015 (7.5%)
Some/completed secondary	123 (44.4%)	5604 (41.4%)
Some/completed tertiary	116 (41.9%)	5862 (43.3%)
**Purpose of visit**		
Existing disease	9 (72.5%)	5906 (44.0%)
New disease	2 (9.9%)	2963 (22.1%)
Pregnancy check	0 (0.0%)	606 (4.5%)
Medical check	0 (0.0%)	2610 (19.4%)
Discomfort, pain, fever	2 (17.6%)	1351 (10.1%)
**Type of visit**		
Outside referral	27 (9.5%)	1454 (10.7%)
Internal referral	18 (6.2%)	532 (3.9%)
Recurring visit	130 (45.6%)	7003 (51.8%)
First visit	110 (38.7%)	4544 (33.6%)
**Facility type**		
Ministry of Health	164 (57.8%)	6156 (45.5%)
EsSalud Insurance	53 (18.7%)	4231 (31.3%)
Armed forces & police	25 (8.5%)	1142 (8.4%)
Private	43 (15.0%)	2004 (14.8%)

Weighted based on sampling weight scaled to full population

Table S3. Full results from multilevel ordinal logistic regression models for 3-category versions of satisfaction (two versions of categorization) and NPS and from multilevel linear regression model for 10-point NPS

	Satisfaction: 3-category[a]		NPS -3 categories		Satisfaction: 3-category[b]		10-point NPS	
	AOR	[95% CI]	AOR	[95% CI]	AOR	[95% CI]	Coeff	[95% CI]
**Contextual**								
% poverty in the district	1.00	1.00, 1.01	1.00	0.99, 1.00	1.00	1.00, 1.01	0.00	0.00, 0.00
Region (Coast)								
Jungle	0.99	0.80, 1.23	1.14	0.87, 1.49	1.03	0.84, 1.28	0.03	-0.11, 0.18
Andean	0.91	0.72, 1.14	1.93	1.44, 2.57	0.93	0.74, 1.17	0.25	0.10, 0.41
Metropolitan Lima	0.81	0.59, 1.10	1.50	1.04, 2.17	0.90	0.67, 1.21	0.20	0.00, 0.40
**Facility-level**								
Facility type (Ministry of Health)								
EsSalud insurance	0.92	0.76, 1.10	0.80	0.63, 1.01	0.95	0.79, 1.14	-0.21	-0.34, -0.09
Armed forces & police	0.86	0.57, 1.29	0.69	0.43, 1.11	0.79	0.54, 1.16	-0.39	-0.64, -0.13
Private	1.42	0.96, 2.12	1.39	0.96, 2.02	0.95	0.69, 1.32	0.09	-0.12, 0.29
Facility level (Primary)								
Secondary	1.02	0.84, 1.23	1.16	0.92, 1.47	1.05	0.87, 1.27	0.11	-0.02, 0.24
Tertiary	1.25	0.94, 1.67	1.75	1.22, 2.51	1.22	0.92, 1.62	0.32	0.13, 0.51
**Individual-level sociodemographic and health**								
Age (<30 years)								
≧ 30 & <45	1.03	0.92, 1.16	0.94	0.85, 1.04	1.05	0.94, 1.17	-0.05	-0.11, 0.01
≧ 45 & <60	1.13	0.99, 1.28	0.99	0.88, 1.10	1.17	1.03, 1.32	0.00	-0.06, 0.07
≧60 years	1.35	1.17, 1.57	1.01	0.89, 1.15	1.33	1.16, 1.53	0.05	-0.02, 0.13
Gender(male)								
Female	0.98	0.90, 1.07	0.99	0.92, 1.07	0.98	0.90, 1.07	0.00	-0.05, 0.04
Wealth (1st, poorest)								
2nd	1.02	0.89, 1.17	1.01	0.89, 1.14	0.95	0.83, 1.08	-0.03	-0.10, 0.04
3rd	1.08	0.93, 1.24	1.02	0.89, 1.15	0.97	0.84, 1.12	-0.07	-0.14, 0.01

	Satisfaction: 3-category[a]		NPS -3 categories		Satisfaction: 3-category[b]		10-point NPS	
	AOR	[95% CI]	AOR	[95% CI]	AOR	[95% CI]	Coeff	[95% CI]
4th	1.16	0.99, 1.35	0.95	0.83, 1.09	1.03	0.88, 1.20	-0.10	-0.18, -0.02
5th (wealthiest)	1.05	0.88, 1.25	0.94	0.80, 1.09	1.06	0.90, 1.26	-0.16	-0.24, -0.07
Education (<Primary)								
Completed primary	1.08	0.89, 1.33	0.97	0.82, 1.16	1.08	0.89, 1.31	0.00	-0.10, 0.11
Some/completed secondary	1.02	0.87, 1.19	0.88	0.76, 1.02	1.10	0.94, 1.28	-0.05	-0.13, 0.03
Some/completed tertiary	0.99	0.83, 1.18	0.80	0.69, 0.94	1.10	0.94, 1.28	-0.09	-0.17, 0.00
Self-rated health (0 - 20)	1.05	1.04, 1.07	1.01	1.00, 1.03	1.05	1.03, 1.06	0.01	0.01, 0.02
Purpose of visit (Existing disease)								
New disease	0.96	0.84, 1.09	1.09	0.97, 1.22	0.93	0.82, 1.06	0.00	-0.06, 0.07
Pregnancy check	1.15	0.92, 1.44	1.00	0.82, 1.21	1.16	0.94, 1.43	-0.03	-0.15, 0.08
Medical check	1.03	0.90, 1.18	1.00	0.89, 1.13	0.99	0.87, 1.13	0.01	-0.06, 0.08
Discomfort, pain, fever	0.81	0.70, 0.95	0.88	0.76, 1.01	0.83	0.71, 0.96	-0.08	-0.16, 0.00
Type of visit (outside referral)								
Internal referral	1.08	0.87, 1.35	0.97	0.79, 1.18	1.04	0.84, 1.30	0.05	-0.06, 0.17
Recurring visit	1.12	0.98, 1.29	1.02	0.89, 1.16	1.03	0.90, 1.19	0.05	-0.02, 0.13
First visit	1.24	1.06, 1.44	0.94	0.81, 1.08	1.17	1.00, 1.36	0.03	-0.05, 0.11
**Patient-reported experience measures (standardized scales)**								
Dignity	1.77	1.60, 1.95	1.05	0.96, 1.16	1.94	1.76, 2.14	0.05	0.00, 0.10
Privacy	0.97	0.92, 1.03	1.10	1.04, 1.16	1.03	0.97, 1.09	0.07	0.04, 0.10
Communication	2.73	2.47, 3.02	2.02	1.83, 2.23	2.81	2.53, 3.13	0.41	0.36, 0.46
Short wait time	1.21	1.15, 1.28	1.06	1.00, 1.11	1.26	1.19, 1.33	0.05	0.02, 0.08
Ease of use	1.27	1.15, 1.41	3.84	3.46, 4.25	1.22	1.10, 1.35	0.83	0.77, 0.88
Provider competence	1.04	0.95, 1.14	0.94	0.86, 1.03	1.08	0.98, 1.19	-0.03	-0.08, 0.02
Timely action	1.11	1.05, 1.17	1.18	1.12, 1.24	1.12	1.06, 1.19	0.13	0.10, 0.16

a: classified “satisfied and very satisfied” into “satisfied” / b: “classified only “very satisfied” into “satisfied”. AOR: Adjusted odds ratio. NPS: Net promoter score.

Table S4: Explained variance by health service quality factors (positive user experience and competence of care and system) from ordinal logistic regression models for 3-category versions of satisfaction (two versions of categorization) and NPS (Pseudo R^2) and from linear regression model for 10-point NPS (R^2)

	Satisfaction: 3 category[a]	NPS: 3 categories	Satisfaction: alternative 3 category[b]	10-point NPS
Model 1	0.033	0.044	0.035	0.077
Model 2	0.199	0.213	0.220	0.372

a: classified “satisfied and very satisfied” into “satisfied” / b: “classified only “very satisfied” into “satisfied”.
Model 1: adjusted for contextual, facility-level, and individual-level factors
Model 2: adjusted for contextual, facility-level, individual-level and patient-reported experience factors
NPS: Net promoter score

Figure S1: Distribution of alternative satisfaction classification by facility recommendation (NPS) responses

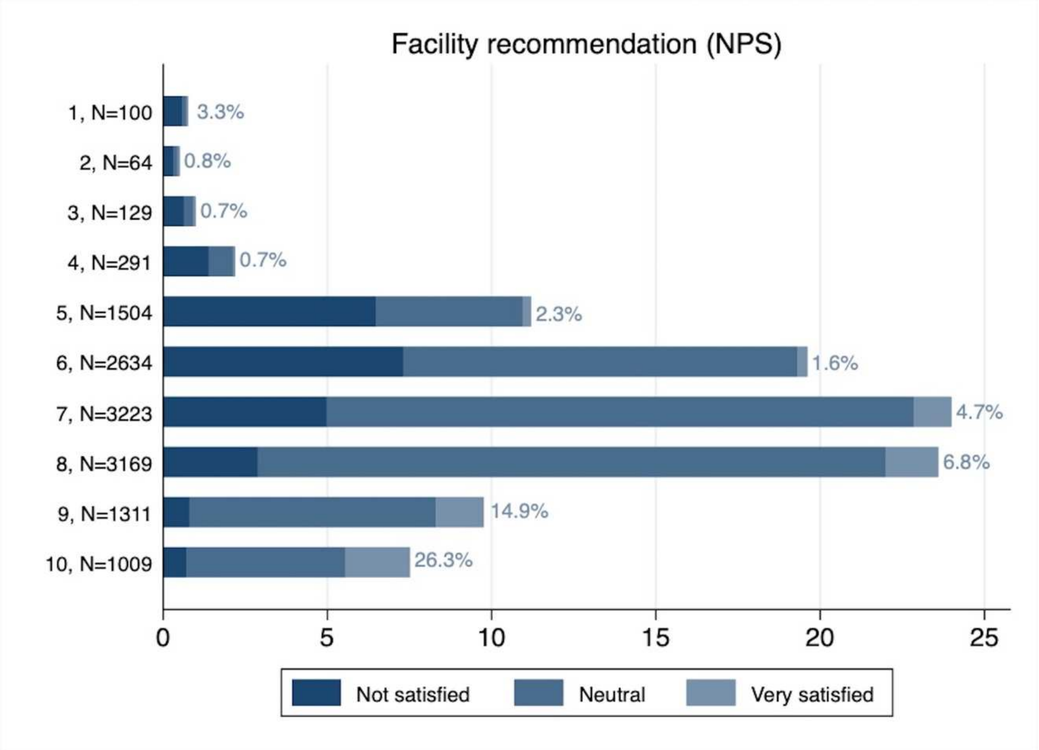

Figure S2: Facility score and 95% confidence interval using alternative satisfaction classification

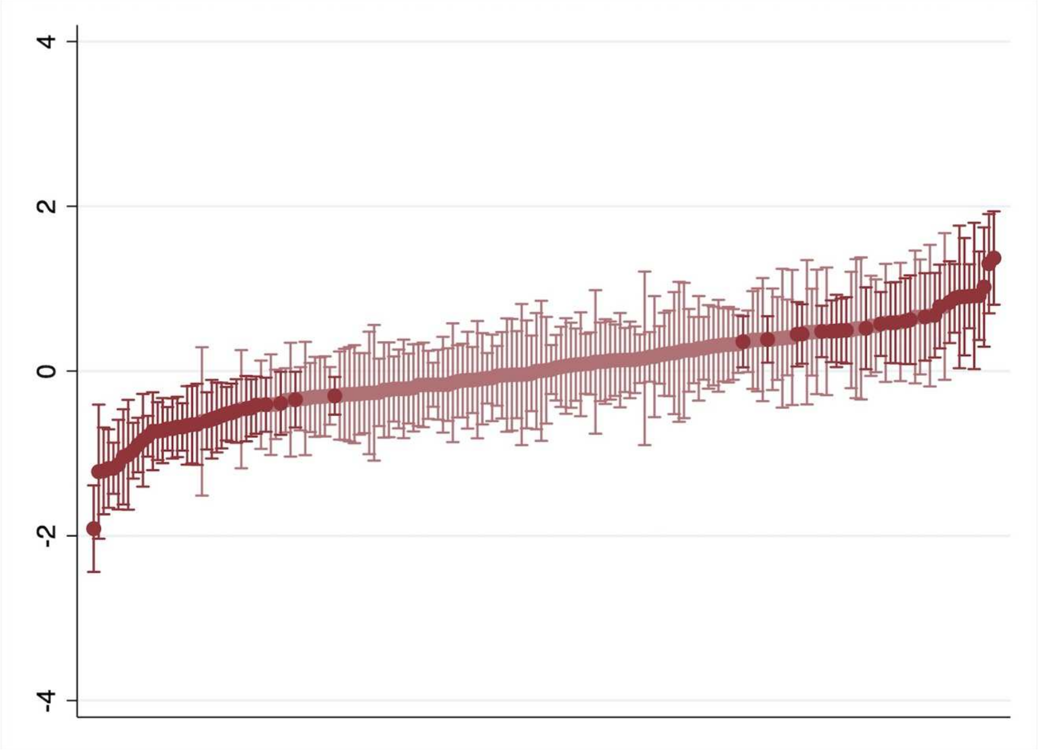

Dark red indicates facilities where the 95% confidence interval excludes the null (0.0). 120 of 184 facilities are indistinguishable from average using this measure (adjusted for case mix).

Intraclass correlation for this version of satisfaction is 0.117 unadjusted, 0.097 adjusted for case mix.

Table S5: Patient ratings within the average category of satisfaction (alternative classification) cat divided by NPS category. N=120 facilities

	Below average NPS (N = 24)	Average NPS (N = 63)	Above average NPS (N = 33)	p-value
	Mean (SD)	Mean (SD)	Mean (SD)	
Dignity	-0.04 (0.23)	0.06 (0.35)	0.21 (0.31)	0.011
Privacy	-0.29 (0.47)	0.11 (0.34)	0.42 (0.32)	<0.001
Communication	-0.12 (0.22)	0.07 (0.28)	0.30 (0.27)	<0.001
Short wait time	-0.33 (0.41)	0.09 (0.40)	0.36 (0.37)	<0.001
Ease of use	-0.34 (0.34)	0.12 (0.31)	0.42 (0.28)	<0.001
Provider competence	-0.04 (0.24)	0.05 (0.34)	0.17 (0.30)	0.037
Timely action	-0.14 (0.39)	0.20 (0.40)	0.44 (0.42)	<0.001

NPS: Net promoter score
